# Interleukin-8 Predicts Fatal Kala-Azar: A Case–Control Study

**DOI:** 10.3390/tropicalmed10090250

**Published:** 2025-09-01

**Authors:** Simone Soares Lima, Débora Cavalcante Braz, Vladimir Costa Silva, Teresinha de Jesus Cardoso Farias Pereira, Líndia Kalliana da Costa Araújo Alves Carvalho, Dorcas Lamounier Costa, Carlos Henrique Nery Costa

**Affiliations:** 1 Department of Maternal and Child Health, Federal University of Piauí, Teresina 64049-550, Piauí, Brazil; dorcas.lc@gmail.com; 2 Postgraduate Program in Biotechnology, Northeast Network of Biotechnology (RENORBIO), Federal University of Piauí, Teresina 64049-550, Piauí, Brazil; lindiakalliana@gmail.com; 3 Pharmacy Course, Federal University of Piauí, Teresina 64049-550, Piauí, Brazil; deborabraz@ufpi.edu.br; 4 Laboratory of Genomic Surveillance and Molecular Biology (LVGBM), Oswaldo Cruz Foundation (FIOCRUZ-PIAUI), Teresina 64000-128, Piauí, Brazil; vladimir.costa@fiocruz.br; 5 Secretary of Education of Piauí, Teresina 64081-110, Piauí, Brazil; teresinhafariasgsc@gmail.com; 6 Intelligence Center for Emerging and Neglected Tropical Diseases (CIATEN), Teresina 64001-020, Piauí, Brazil; chncosta@gmail.com; 7 Laboratory of Leishmaniasis, Natan Portella Institute of Tropical Medicine, Teresina 64001-020, Piauí, Brazil; 8 Department of Community Medicine, Federal University of Piauí, Teresina 64001-020, Piauí, Brazil

**Keywords:** visceral leishmaniasis, prognosis, cytokines, inflammation, mortality

## Abstract

Kala-azar is associated with case-fatality rates as high as 10% in certain regions. Early identification of mortality biomarkers can significantly reduce this risk. This study, strengthened by a relatively high number of kala-azar-related deaths, aimed to identify serum cytokines as predictive biomarkers of fatal kala-azar. We compared 48 deceased patients with kala-azar to 42 survivors. The concentrations of IL-1β, IL-6, IL-8, IL-10, IL-12, and tumor necrosis factor-α (TNF-α) were measured by flow cytometry. Cytokine levels were compared between groups using the Wilcoxon rank-sum test. Receiver operating characteristic (ROC) analysis, coupled with Youden’s index, defined the optimal diagnostic threshold. Upon admission, IL-8 concentrations were substantially higher in deceased kala-azar patients (median 76.5 pg/mL [IQR 35.2–242.4 pg/mL]) than in survivors (median 26.4 pg/mL [IQR 15.1–47.7 pg/mL]; *p* < 0.0001). ROC analysis identified 49.3 pg/mL as the optimal cutoff. When rounded to the clinically convenient value of 50 pg/mL, IL-8 predicted a fatal outcome with an area under the curve of 0.75, sensitivity of 70.8%, and specificity of 76.2%. In contrast, IL-1β, IL-6, IL-10, IL-12, and TNF-α showed no significant prognostic utility. Our findings suggest that IL-8 levels equal to or greater than 50 pg/mL are a reliable predictor of fatal kala-azar.

## 1. Introduction

The healthcare professional’s perception of disease severity at the first contact with the patient is what determines the level of care, with life-threatening conditions being one of the leading indicators of this level. Early evaluation using biomarkers improves the accuracy of clinical assessment. It may help reduce mortality rates, particularly in the case of kala-azar, a disease responsible for approximately 5482 deaths per year [1]. 

Kala-azar, or visceral leishmaniasis, is associated with case-fatality rates as high as 10% in certain regions [2,3]. Brazil accounts for 91% of kala-azar in the Americas, approximately 1461 cases [4]. The high incidence of cases of coinfection of kala-azar with human immunodeficiency virus (HIV), especially in endemic areas, makes kala-azar an important public health problem [4].

Kala-azar is caused by *Leishmania infantum* in the Middle East, Central Asia, South America, the Mediterranean Basin, and *L. donovani* in South Asia and East Africa [5]. Almost all transmission in the New World is by the bite of female insects of the genus *Lutzomyia* [6]. The most common symptoms of kala-azar are fever, splenomegaly, hepatomegaly, weight loss, and cutaneous mucocutaneous pallor [7,8,9]. Bacterial infections and bleeding are associated with fatal outcomes [10,11,12]. 

In symptomatic infection by *Leishmania* spp., the innate immune response, characterized by high inflammation, may help predict clinical progression [12]. In this context, pretreatment serum levels of interferon-gamma (IFN-γ), tumor necrosis factor-α (TNF-α), IL-4, IL-6, IL-8, IL-10, and IL-27 are elevated in Brazilian individuals with untreated kala-azar [13,14,15]. Conversely, cytokine levels in asymptomatic infection are similar to those detected in cured [14] or uninfected individuals [13].

Similar data have been reported in other countries. In Sudan, individuals with kala-azar have high levels of IFN-γ, TNF-α, IL-4, IL-6, IL-10, IL-12, and IL-17A [16]. In Kenya, the authors reported elevated levels of IFN-γ, IL-5, IL-6, IL-10, IL-12, IL-17A, and IL-27 in symptomatic cases compared to healthy controls [17]. In Iran, higher levels of IFN-γ, IL-1β, IL-10, IL-12, and IL-17 were evident in symptomatic cases [18,19]. On the other hand, patients from Bangladesh with kala-azar had lower levels of IL-12 associated with elevated levels of IL-8 and IL-10 [20]. 

These differences in cytokine levels according to the clinical presentation of kala-azar are also reproduced in kala-azar and HIV coinfection. Asymptomatic individuals have cytokine levels similar to those of healthy controls. In contrast, in symptomatic cases coinfected with HIV and kala-azar, elevated serum levels of IL-6, IL-10, and IL-17A were identified compared to healthy and asymptomatic controls [21].

The exacerbated profile of pro- and anti-inflammatory cytokines in patients with kala-azar appears related to disease worsening, particularly in the levels of IFN-γ, IL-6, IL-8, and IL-10 [15,17,22]. Even in non-severe kala-azar, we identified that levels of IL-6, IL-8, and IL-10 are promising biomarkers of early response to treatment [23]. In Brazilian individuals, high levels of IL-6, L-8, and IFN-γ were associated with death from kala-azar, higher levels of L-8 and IFN-γ were evident in cases with hemorrhage, and IL-6 and IFN-γ were associated with increased coagulation markers [22].

However, a study in Kenya showed lower levels of IFN-γ and high levels of IL-10 in patients with severe kala-azar [17]. It is worth noting that IL-10 levels are associated with a higher parasite load [24], whereas high levels of IL-12 are correlated with lower parasitemia [25]. The results presented highlight the importance of investigating the predictive power of inflammatory and regulatory cytokines as prognostic biomarkers in kala-azar. 

Although valuable previous publications have evaluated cytokines as biomarkers of evolution or outcome in kala-azar, our work advances this field by analyzing cytokines with an optimal cutoff point determined to distinguish individuals with severe kala-azar at high risk of death from those with a greater chance of survival. This case–control study is supported by the high proportion of deaths related to kala-azar compared to surviving controls, the determination of an optimal cutoff point for the selected cytokines as biomarkers of severity in kala-azar, and the analysis of the discriminatory capacity of these immunological markers in predicting severe kala-azar.

The use of immunological biomarkers at disease onset or at the first clinical evaluation enables the early identification of individuals at risk of severe kala-azar, and consequently, the timely implementation of therapeutic measures to improve survival. Therefore, we compared pretreatment levels of IL-1β, IL-6, IL-8, IL-10, IL-12, and TNF-α between kala-azar patients who survived and those who died, in order to assess their potential as mortality biomarkers.

## 2. Materials and Methods

### 2.1. Study Design and Setting

We conducted a case–control study in Piauí, Brazil, an area endemic for epidemic outbreaks [26]. The research center was the Nathan Portella Institute of Tropical Diseases (IDTNP), the reference hospital for treating kala-azar in Teresina, Piauí. 

The participants were selected from a cohort that reported 1009 individuals with kala-azar confirmed by clinical and laboratory criteria over a five-year period, of whom 80 died from kala-azar during the five years. The deceased individuals were classified as cases, and the survivors as controls.

### 2.2. Inclusion Criteria and Follow-Up

The inclusion criteria were consecutively admitted, newly diagnosed, and untreated symptomatic kala-azar patients proven by molecular, serological, or parasitological diagnostic techniques. There were no restrictions on sex or age and no exclusion criteria. The attending physician and one of the researchers followed the participants prospectively from admission to the date of discharge or fatal outcome.

The randomization of participants was performed at a 1:1 ratio of deceased patients to survivors using Stata/BE 15.1 software. We randomized 90 participants, comprising 48 (53.3%) deceased patients (cases) and 42 (46.7%) survivors (controls). 

HIV infection was not a criterion for randomization. The inclusion of participants with kala-azar who were also infected with HIV occurred unintentionally. 

### 2.3. Serum Cytokines 

Serum samples collected prior to treatment were stored at −20 °C until cytokine analysis was conducted. The levels of IL-1β, IL-6, IL-8, IL-10, TNF-α, and the interleukin-12 p70 heterodimer (IL-12) were measured by flow cytometry using the Human Inflammatory Cytokines CBA Kit (BD Biosciences, San Jose, CA) on a BD FACS Canto™ instrument (Becton Dickinson, USA) following the manufacturer’s instructions [27], and the levels of clinical and laboratory markers of kala-azar activity were analyzed.

### 2.4. Clinical and Laboratory Variables 

Direct medical interviews or clinical records were obtained using demographic, clinical, and laboratory variables. Haematologic analysis of peripheral blood, kidney and liver function tests, comorbidities, and complications was also evaluated as part of the routine investigation of patients with suspected kala-azar.

### 2.5. Statistical Analysis

The analysis was conducted using Stata Now/BE 15.1 software. Dichotomous variables were assessed with Fisher’s exact or Chi-square tests [28]. We determined the medians and interquartile ranges of cytokine levels in both deceased patients with kala-azar and survivors [29]. Wilcoxon rank-sum tests were then employed to compare these median cytokine levels between non-survivors and survivors [28]. Additionally, we used the Wilcoxon rank-sum test to compare cytokine levels between patients with and without HIV coinfection, evaluating the impact of HIV on cytokine distribution across these groups.

We analyzed the sample power using a two-sample, two-tailed *t*-test with unequal variances (Welch’s *t*-test) [30] based on the observed differences in the means and standard deviations of IL-8, the main biomarker in this study, between the 42 individuals with kala-azar who survived (mean = 176; standard deviation [SD] = 103) and the 48 individuals who died (mean = 267; SD = 75). We concluded that, in this sample of 90 individuals with kala-azar, it is possible to detect the difference in IL-8 means with a statistical power of 99.7%, assuming a significance level of α = 0.05.

To evaluate the potential of IL-1β, IL-6, IL-8, IL-10, IL-12, and TNF-α as predictive biomarkers for identifying fatal cases of kala-azar, we conducted receiver operating characteristic (ROC) curve analysis [31]. We first assessed the cytokine levels in their continuous form against a dichotomous outcome (fatal versus non-fatal kala-azar) to determine their discriminatory power. We then established optimal cutoff points using the Youden index [31,32].

Only the cytokines that yielded an area under the curve (AUC) greater than 0.70 or less than 0.30, along with sensitivity and specificity equal to or greater than 70%, were selected for further analysis. 

Cytokines that met the criteria related to AUC, sensitivity, and specificity were transformed into dichotomous variables based on their Youden-derived cutoff points. In this categorization, a value of 1 (one) was assigned to individuals with cytokine concentrations equal to or greater than the established cutoff point. In contrast, the value 0 (zero) was assigned to those with concentrations below this limit. Additionally, we employed the DeLong test to compare the areas under the curve (AUCs) [33].

### 2.6. Ethical Approval 

The study was approved by the Human Research Ethics Committee of the Federal University of Piauí (CAAE 0116.0.045.203-05, date 14 December 2005) and conducted in accordance with the ethical standards outlined in the Declaration of Helsinki. All patients provided written informed consent.

## 3. Results

### 3.1. Study Population 

A total of 90 individuals with kala-azar were included in the study, comprising 48 deceased patients (53.3%) and 42 survivors (46.7%). The majority were male (73.3%) and adults (53.3%), with a mean age of 29.5 years (range: 5.7 months to 78 years). Among the nineteen participants living with HIV or AIDS, 16 (84.2%) were in the deceased group (*p* = 0.002). Individuals aged 60 years or older, as well as those presenting with hemorrhagic or infectious complications, were significantly more frequent among the deceased (*p* < 0.05) (Table 1).

The median and mean durations of fever until hospital admission were 30 and 60 days, respectively. The most frequent clinical manifestations were fever (90.0%), mucocutaneous pallor (89.0%), fatigue (84.4%), splenomegaly (81.1%), weight loss (77.8%), and hepatomegaly (56.2%). The mean length of the spleen was 6 cm, with a maximum length of 20 cm, and the mean length of the liver was 3 cm, with a maximum length of 14 cm. These signs and symptoms were similar between deceased patients and survivors (*p* > 0.05). 

However, some laboratory data were statistically different between survivors and deceased patients. The last group exhibited significantly lower mean hemoglobin levels (7.4 g/dL vs. 8.1 g/dL; *p* = 0.032), reduced mean albumin levels (2.9 g/dL vs. 3.3 g/dL; *p* = 0.043), and elevated mean creatinine levels (1.8 mg/dL vs. 0.9 mg/dL; *p* = 0.008) compared to survivors. 

Conversely, the overall mean leukocyte count was 3183.5/mm^3^, with no statistically significant differences between the deceased and survivor cohorts (3453.7/mm^3^ vs. 2867.2/mm^3^, *p* = 0.3438). Similarly, the overall mean neutrophil count was 1426.6/mm^3^ (1751.5/mm^3^ vs. 1037.0/mm^3^, *p* = 0.9132), the mean platelet count was 100,800/mm^3^ (92,818/mm^3^ vs. 110,146/mm^3^, *p* = 0.1991), the mean AST concentration was 82.2 U/L (101.0 U/L vs. 60.2 U/L, *p* = 0.0835), and the mean ALT concentration was 53.2 U/L (51.0 U/L vs. 55.7 U/L, *p* = 0.6292).

### 3.2. Serum Cytokine Levels in Survivors and Non-Survivors with Kala-Azar 

We performed cytokine analysis on all study participants with the following minimum and maximum values detected: IL-1β (0 to 688.7 pg/mL), IL-6 (1.1 to 7293.1 pg/mL), IL-8 (2.7 to 4101.0 pg/mL), IL-10 (0 to 238.2 pg/mL), IL-12 (0 to 35.1 pg/mL), and TNF-α (0 to 59.3 pg/mL). 

In the analysis of cytokine correlations with age, sex, nutritional status (defined by body mass index adjusted for age and sex), and disease duration, we observed negative correlations of age with IL-10 (ρ = –0.23, *p* = 0.0317) and TNF-α (ρ = –0.27, *p* = 0.0108), as well as a weak positive correlation of IL-12 with disease duration (ρ = 0.32, *p* = 0.0027). IL-6, IL-8, and IL-1β showed no statistically significant correlations with these parameters.

**Figure 1:**
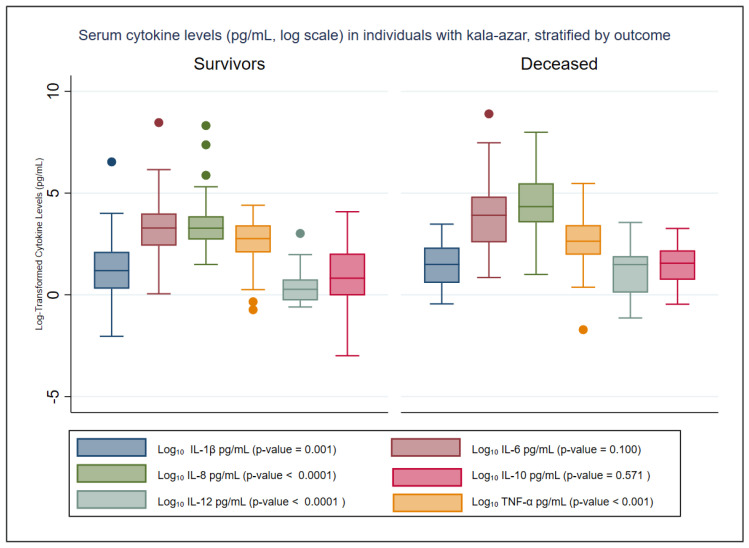
Distribution of pretreatment serum cytokine levels (pg/mL, Log Scale) in kala-azar patients stratified by outcome. This figure illustrates the distribution of serum cytokine levels measured prior to the initiation of specific anti-*Leishmania* treatment in patients with kala-azar, stratified into survivors and non-survivors. Values are expressed in pg/mL and were log_10_-transformed to improve visualization of data variability.Colored dots indicate outliers beyond 1.5 times the interquartile range.

The distribution of pre-treatment cytokine levels among survivors and deceased kala-azar patients is illustrated in Figure 1 and Table 2. Reference values for these cytokines, based on data from healthy blood donors reported by Kildey et al. (2014), are also included in Table 2 for comparison [34].

Table 2 presents the mean and median (interquartile range, IQR) cytokine concentrations in patients with kala-azar, stratified by outcome (survivors vs. deceased). At hospital admission, IL-8 levels were statistically significantly higher in deceased patients (*p* < 0.0001), while IL-1β, IL-12, and TNF-α levels were significantly lower (*p* < 0.002). The concentrations of IL-6 and IL-10 did not differ significantly between the groups, although IL-6 was almost two times higher among those who had recently died (Table 2). 

In a subgroup composed exclusively of individuals co-infected with kala-azar and HIV, fatal cases showed significantly lower mean levels of IL-1β (0.8 pg/mL vs. 1.4 pg/mL; *p* = 0.0280), IL-12 (0.0 pg/mL vs. 1.8 pg/mL; *p* < 0.0001), and TNF-α (0.04 pg/mL vs. 0.8 pg/mL; *p* = 0.0107). However, no significant difference was detected in individuals who died compared to survivors in the mean levels of IL-6 (118.3 pg/mL vs. 48.4 pg/mL; *p* = 0.4338), IL-8 (160.2 pg/mL vs. 29.2 pg/mL; *p* = 0.1461), and IL-10 (15.1 pg/mL vs. 18.0 pg/mL; *p* = 0.7372).

### 3.3. Diagnostic Performance of Serum Cytokines in Predicting Fatal Kala-Azar

Table 3 presents the diagnostic potential of IL-1β, IL-6, IL-8, IL-10, IL-12, and TNF-α in predicting fatal kala-azar. Among the cytokines evaluated, IL-8 emerged as the most promising biomarker for predicting mortality in individuals with kala-azar. It showed good discriminative power, with an AUC of 0.75 and a 95% confidence interval (95% CI) of 0.65 to 0.86. At its optimal cutoff of 49.3 pg/mL, IL-8 achieved a sensitivity of 70.8% and a specificity of 76.2%. Conversely, IL-6 exhibited low discriminatory power, with an AUC of 0.60 (95% CI: 0.48–0.72). Even more notably, IL-1β (AUC: 0.31, 95% CI: 0.20–0.42), IL-10 (AUC: 0.47, 95% CI: 0.34–0.59), IL-12 (AUC: 0.30, 95% CI: 0.17–0.37), and TNF-α (AUC: 0.29, 95% CI: 0.18–0.40) all presented AUC values below 0.5. 

A reliable cutoff point could not be determined for IL-1β, IL-12, and TNF-α due to a high frequency of zero values, which limited the performance of logistic regression analysis.

### 3.4. Serum Cytokine Profiles by HIV Coinfection Status

As shown in Table 4, we evaluated cytokine levels (means, medians, and interquartile ranges) in kala-azar patients stratified by HIV coinfection status. This analysis was prompted by the observation that 33.3% of patients who died were HIV coinfected. Our findings indicate that IL-6, IL-8, and IL-10 concentrations were similar in HIV-coinfected and non-HIV-coinfected kala-azar patients (*p* > 0.05). In contrast, IL-1β, IL-12, and TNF-α levels were significantly lower in HIV-coinfected patients (*p* < 0.05).

### 3.5. Diagnostic Performance of Dichotomized IL-8 in Predicting Fatal Kala-Azar

IL-8 was the only cytokine that met all predefined criteria to be considered a biomarker for fatal kala-azar, demonstrating an AUC greater than 0.70, with both sensitivity and specificity exceeding 70%, as shown in Table 3.

**Figure 2:**
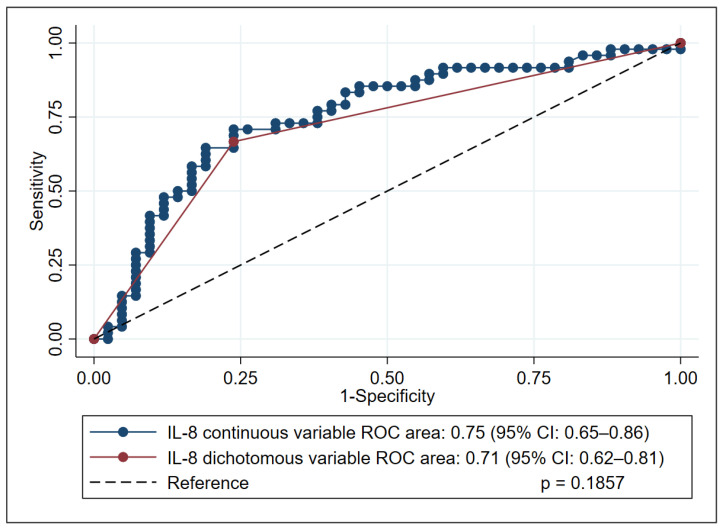
Receiver operating characteristic (ROC) curve analysis of interleukin-8 (IL-8) performance in predicting mortality in kala-azar patients, comparing continuous and dichotomized forms. This figure illustrates the predictive performance of IL-8 (in both its continuous and dichotomized forms) for mortality in patients with kala-azar. The comparison of their respective areas under the curve (AUCs) was performed using DeLong’s test.

In the subsequent analysis, IL-8 was dichotomized using a cutoff of 50 pg/mL, a clinically convenient threshold that closely approximates the Youden-derived optimum of 49.3 pg/mL. With this binary classification, IL-8 maintained robust discriminatory performance, producing an AUC of 0.71 (95% CI, 0.62–0.81), sensitivity of 76.2% (95% CI: 60.5–87.9), specificity of 66.7% (95% CI: 51.6–79.6), positive predictive value (PPV) of 66.7% (95% CI: 51.6–79.6), and negative predictive value (NPV) of 76.2% (95% CI: 60.5–87.9) and demonstrating no loss of information relative to the continuous-variable analysis (*p* = 0.1857) (Figure 2).

In the subgroup of 71 individuals with kala-azar without HIV infection, we identified an AUC of 0.74 (95% CI: 0.64–0.85), sensitivity of 71.9% (95% CI: 53.3–86.3), specificity of 76.9% (95% CI: 60.7–88.9), PPV of 71.9% (95% CI: 53.3–86.3), and NPV of 76.9% (95% CI: 60.7–88.9). In this population, significantly higher IL-8 levels were confirmed in fatal cases compared to survivors (320.4 pg/mL versus 187.8 pg/mL, *p* < 0.0001).

### 3.6. Influence of HIV Coinfection on IL-8 Discriminatory Power

To assess whether HIV coinfection affects the performance of IL-8 as a biomarker for fatal outcomes in kala-azar, we conducted separate AUC analyses for HIV-coinfected and non-coinfected patients. No significant differences were observed between the groups, as shown in Table 5.

## 4. Discussion

In our study, the proportion of deceased patients with kala-azar (cases) was 1.1:1 relative to survivors (controls). This balanced ratio of deaths to survivors aimed to optimize the identification of cytokines as biomarkers predictive of an unfavorable prognosis. Furthermore, we randomized cases from the same healthcare facility and hospitalized them concurrently with the controls. Based on the selection process, these cases seem to be representative of the overall study population.

We employed ROC curve analysis to thoroughly assess the potential of IL-1β, IL-6, IL-8, IL-10, IL-12, and TNF-α as predictive biomarkers for identifying fatal cases of kala-azar. Our findings indicate that measuring IL-8 at the initial clinical encounter can effectively predict fatal outcomes in kala-azar patients, demonstrating good sensitivity and specificity. Notably, IL-8 maintained consistent predictive performance in both HIV-coinfected and non-coinfected individuals. With a Youden-derived cutoff point of approximately 50 pg/mL, IL-8 showed meaningful discriminatory power in identifying individuals at increased risk of death.

Since we are evaluating biomarkers related to fatal outcomes in kala-azar, it is expected that cytokines already known to be implicated in the pathogenesis of this disease will emerge as good predictors. IL-12 has been conceptually associated with better clinical outcomes in kala-azar [25]. Similarly, IL-1β and TNF-α have been reported as cytokines with contradictory roles, being sometimes protective and at other times pathogenic [35,36,37]. IL-10, while acting as an immunosuppressive mediator that facilitates parasite persistence, may also modulate disease manifestations [24]. IL-6 and IL-8 have consistently been identified as pathogenic [15,22,38], yet, in our study, only IL-8 emerged as a robust biomarker for severe or fatal kala-azar, although IL-6 levels were also elevated in fatal cases.

IL-8 is a pro-inflammatory chemokine secreted chiefly by activated monocytes, macrophages, and endothelial cells. It functions as the principal chemoattractant for neutrophils and a potent regulator of their activation [39,40]. 

Kala-azar, characterized by an exacerbated inflammatory immune response and associated coagulopathy, shares important pathophysiological features with sepsis, although with a more protracted clinical course [12]. Given these similarities, it is plausible that comparable mechanisms may also operate in kala-azar, although this has not yet been directly demonstrated.

In this context, similar to observations in sepsis, endothelial cell injury leads to the secretion of inflammatory cytokines, including IL-8 [41]. This chemokine can amplify inflammation through endothelial cells in a positive feedback loop [42]. IL-8 recruits neutrophils to organs parasitized by *Leishmania*, which in turn secrete cytokines such as IL-6, a mediator associated with disease severity in kala-azar [15,22]. Consequently, the inflammatory response is intensified, contributing to the increased pathogenicity of the disease. An experimental study confirmed the pathogenic role of IL-8 as a neutrophil chemoattractant, demonstrating that its neutralization significantly reduced lung injury [43].

It is worth noting that other neutrophil chemoattractant factors may be present during an infectious process, as suggested by an experimental study in which IL-8 neutralization did not completely prevent neutrophil influx at the inflammatory site [43]. One candidate for this explanation is the complement fragment C5a, which contributes to the expression of the IL-8 gene [44]. In this context, studies in sepsis models have identified C5a as a crucial inflammatory mediator that enhances IL-8 production [45]. The relationship of IL-8, C3a, and C5a with inflammation, pathogenicity, and target-organ lethality, as well as the positive correlation between these anaphylatoxins and IL-8, has been confirmed in the lungs of patients infected with coronaviruses [46].

IL-8 concentrations are elevated in severe kala-azar [22]. Therefore, measuring IL-8 early in the disease process through venipuncture makes this chemokine a minimally invasive biomarker for identifying fatal kala-azar and guiding clinical intervention as early as possible.

Prior studies have already indicated the importance of IL-8 in kala-azar. Significantly elevated serum levels of IL-6, IL-8, and IFN-γ have been reported in patients with fatal outcomes [22]. It was observed that symptomatic individuals exhibited higher IL-8 levels compared to asymptomatic ones [20] and that this chemokine served as an early marker of treatment response in patients without severe signs [23]. Additionally, IL-8 has been independently associated with hemorrhage, a key marker of disease severity [22]. 

This novel finding aligns with the broader established role of IL-8 as a valuable prognostic biomarker across various inflammatory and infectious conditions [47,48,49,50,51]. For example, high IL-8 levels have been correlated with increased multiple organ failure, sepsis, and mortality in pediatric patients with severe burns [48]. Furthermore, IL-8 has been proven to be a sensitive and specific biomarker for neonatal infection, and its association with severity has been established [49]. Among adults with sepsis, plasma IL-8 levels measured within the first 24 h of diagnosis can predict disease severity and 28-day mortality [51]. Conversely, in children younger than ten years with septic shock, serum IL-8 levels below 220 pg/mL predict 28-day survival with 95% accuracy [52]. 

Our results underscore the role of specific cytokines as opposing biomarkers in kala-azar. The chemokine IL-8 was linked to fatal outcomes, while the levels of IL-1β, IL-12, and TNF-α were found to be lower in patients who ultimately died. Although levels of IL-6 and IL-10 did not show significant differences between the groups, pre-treatment IL-6 levels were twice as high in individuals who died.

IL-6 is a pro-inflammatory cytokine that also demonstrates immunosuppressive effects by stimulating the production of IL-10 [53,54], thereby reducing resistance to intracellular infections. IL-6 contributes to disease severity and mortality in kala-azar [15,22], thereby contributing to the pathogenesis of coagulopathy in this condition [22].

Although the finding of higher pre-treatment IL-1β, IL-12, and TNF-α levels in survivors suggests they serve as protective biomarkers in individuals with kala-azar, the accuracy was not sufficient for adequate diagnostic utility. These cytokines also did not show good sensitivity as biomarkers of activity in untreated and non-severe kala-azar, as we suggested in a previous study [23].

Concentrations of IL-1β and TNF-α are often elevated in individuals with symptomatic kala-azar [13,15,19]. At the same time, inflammasome-derived IL-1β stimulates the synthesis of nitric oxide, a leishmanicidal agent produced by macrophages [35]. While the protective actions of TNF-α include macrophage activation, granuloma formation in experimental models [36], and stimulation of IFN-γ production, the main cytokine responsible for macrophage activation, no relationship with parasitic load has been demonstrated in kala-azar [55].

Lower levels of IL-12 in fatal kala-azar may be justified by its role as a cytokine that activates CD4+ type 1 helper T cells, stimulating natural killer (NK) cells to produce IFN-γ [56], in addition to being associated with lower parasitemia in kala-azar [25]. Reports of congenital deficiencies of IL-12p40 or the IL-12 receptor, which increase susceptibility to recurrent Leishmania infections, further reinforce this idea [57].

The concentrations of IL-1β, IL-12, and TNF-α were significantly decreased in individuals coinfected with kala-azar and HIV compared to the values detected in cases of kala-azar without HIV infection. No differences were observed for IL-6, IL-8, and IL-10 levels.

Although the number of individuals with kala-azar co-infected with HIV represented only 20% of the study participants, the results detected in this subgroup pointed in the same direction, with lower levels of the potentially protective cytokines IL-1β, IL-12, and TNF-α in fatal cases. It is noteworthy that IL-6 and IL-8 levels were approximately twice as high in individuals without HIV compared to those detected in kala-azar and HIV co-infection, suggesting that HIV infection may attenuate the exaggerated inflammatory response, which probably occurs due to damage to the Th1 response. 

Recent publications involving Brazilian individuals with kala-azar and HIV have reported lower levels of IL-10 [22,25] and IFN-γ [22] in HIV and kala-azar co-infected individuals compared to those with kala-azar without HIV. In our study, IL-10 levels in individuals with HIV and kala-azar were almost half of the values found in individuals without HIV, but without statistical significance (*p* = 0.0551). 

IL-10 is a regulatory cytokine commonly associated with a weaker response to Leishmania infection, as demonstrated by its association with higher parasite burdens [24], which may justify higher levels in populations with a greater number of deaths. 

Similar to what is observed in kala-azar without HIV, patients with HIV and kala-azar who are symptomatic present higher levels of IL-17A, IL-6, and IL-10 compared to co-infected individuals without symptoms [21]. IL-6 and IL-10 are increased in kala-azar and HIV co-infection, while IL-6 is associated with severity signs and with the fatal outcome [38], similar to what is described in individuals with kala-azar without HIV [22].

Our findings showed that patients who progressed to death had significantly lower levels of IL-1β, IL-12, and TNF-α, similar to those identified in individuals coinfected with HIV. Notably, 33% of the fatal cases occurred in HIV-positive patients, suggesting that the reduced cytokine levels may, at least in part, be due to the immunomodulatory effects associated with HIV coinfection. However, these cytokines did not exhibit acceptable discriminatory performance in ROC curve analysis.

Elevated levels of IL-1β, IL-12, and TNF-α may have a protective effect in individuals with kala-azar; however, in the present study, these cytokines did not demonstrate adequate diagnostic utility. In summary, our findings indicate that a serum IL-8 concentration of 50 pg/mL or greater, measured at the initial clinical encounter, can effectively distinguish between fatal and non-fatal cases of kala-azar with high sensitivity and specificity, thereby aiding clinical decisions at the point of care. Therefore, early quantification of IL-8 emerges as a promising tool for risk stratification. By facilitating the prompt identification of critically ill patients, this strategy enables clinicians to intensify surveillance and institute early, targeted interventions and measures that could lower the case-fatality rate of kala-azar, which stubbornly remains near 10 % even when appropriate therapy is initiated without delay.

## 5. Conclusions

Our data demonstrate that serum IL-8 levels equal to or above 50 pg/mL at the initial clinical encounter are a reliable predictor of fatal kala-azar.

## Figures and Tables

**Table 1:** Demographic and clinical characteristics of kala-azar patients by outcome (survivors vs. deceased).

Variables	Survivors (n = 42) n (%)	Deceased (n = 48) n (%)	Total (n = 90) n (%)	*p*-Value
**Sex**				0.924 ^a^
Male	31 (73.8)	35 (72.9)	66 (73.3)	
Female	11 (26.2)	13 (27.1)	24 (26.7)	
**Age groups**				
Child (age < 18 years)	23 (54.8)	9 (18.7)	32 (35.6)	<0.0001 ^a^
Adult (18–59 years)	18 (42.8)	30 (62.5)	48 (53.3)	0.06 ^a^
Elderly (≥60 years)	1 (2.4)	9 (18.8)	10 (11.1)	0.014 ^a^
**Comorbidities**				
HIV coinfection	3 (7.1)	16 (33.3)	19 (21.1)	0.002 ^b^
**Complications during hospitalization ^c^**				
Bacterial infection	12 (28.6)	27 (56.2)	39 (43.3)	0.008 ^a^
Hemorrhage	1 (2.4)	22 (45.8)	23 (25.6)	<0.0001 ^b^

Abbreviations: HIV, human immunodeficiency virus; n, absolute number; %, relative frequency. ^a^ Chi-squared test; ^b^ Fisher’s Exact test. ^c^ Patients may have presented with more than one complication during hospitalization.

**Table 2:** Pretreatment cytokine levels (pg/mL) in survivors and deceased patients with kala-azar.

Cytokine	Survivors (n = 42)		Deceased (n = 48)		Total (n = 90)		*p*-Value *	Reference ^1^
	Mean	Median (IQR)	Mean	Median (IQR)	Mean	Median (IQR)		Median (IQR)
IL-1β	22.7	2.5 (1.0–6.6)	3.6	0.0 (0.0–4.0)	12.5	1.7 (0.0 –6.0)	0.001	0.2 (0.0–3.7)
IL-6	161.3	26.7 (11.2–54.7)	334.0	49.9 (13.1–125.1)	253.4	30.1 (11.4–77.1)	0.100	0.0 (0.0–0.0)
IL-8	176.5	26.4 (15.1–47.7)	267.0	76.5 (35.2–242.4)	224.8	46.6 (21.9–120.5)	<0.0001	0.0 (0.0–0.0)
IL-10	21.4	15.9 (8.0–30.6)	27.4	13.1 (4.8–29.5)	24.6	13.8 (5.6–30.6)	0.571	0.0 (0.0–0.0)
IL-12	1.8	0.9 (0.0–2.0)	1.5	0.0 (0.0–0.0)	1.6	0.0 (0.0–1.1)	<0.0001	0.0 (0.0–0.0)
TNF-α	6.1	1.7 (0.3–6.5)	2.5	0.0 (0.0–2.4)	4.2	0.8 (0.0–3.9)	<0.001	0.0 (0.0–0.0)

Abbreviations: IL, interleukin; IL-1β, interleukin-1 beta; IQR, interquartile range; pg/mL, picograms per milliliter; TNF-α, tumor necrosis factor-alpha. Notes: * *p*-values were calculated using the Wilcoxon rank-sum test to compare cytokine levels between survivors and patients who died from kala-azar. Bolded values indicate statistical significance (*p* < 0.05). Values of 0.0 indicate concentrations below the limit of detection. ^1^ Reference values from healthy blood donors are based on Kildey et al. (2014) [34].

**Table 3:** Diagnostic performance of serum cytokines (IL-1β, IL-6, IL-8, IL-10, IL-12, and TNF-α) in predicting death in patients with kala-azar (n = 90).

Cytokine	Optimal Cutoff (pg/mL)	Sensitivity (%)	Specificity (%)	AUC (95 % CI)
IL-1β	0.6	56.3	88.1	0.31 (0.20–0.42)
IL-6	49.5	52.1	73.8	0.60 (0.48–0.72)
IL-8	49.3	70.8	76.2	0.75 (0.65–0.86)
IL-10	45.3	16.7	92.9	0.47 (0.34–0.59)
IL-12	0.3	83.3	69.0	0.30 (0.17–0.37)
TNF-α	0.0	62.5	85.7	0.29 (0.18–0.40)

Abbreviations: AUC: area under the receiver operating characteristic curve; CI: confidence interval; IL, interleukin; IL-1β, interleukin-1 beta; TNF-α, tumor necrosis factor-alpha. Notes: The sample size (n = 90) represents the total number of patients with valid cytokine measurements included in the analysis for each respective cytokine. Optimal cutoff points were determined using the Youden Index. Sensitivity represents the proportion of fatal cases correctly identified above the cutoff. Specificity represents the proportion of survivors correctly identified below the cutoff.

**Table 4:** Serum cytokine levels (pg/mL) in individuals with kala-azar, stratified by HIV coinfection status.

Cytokine	Kala-Azar without HIV (n = 71)		Kala-Azar with HIV (n = 19)		Total (n = 90)		*p*-Value *
	Mean	Median (IQR)	Mean	Median (IQR)	Mean	Median (IQR)	
IL-1β	15.6	2.3 (0.7–7.0)	0.9	0.0 (0.0–0.7)	12.5	1.7 (0.0–6.0)	0.0002
IL-6	292.5	29.8 (11.7–77.1)	107.2	34.7 (10.5–129.4)	253.4	30.1 (11.4–77.1)	0.9404
IL-8	247.6	45.5 (19.2–124.1)	139.5	53.4 (25.8–104.5)	224.8	46.6 (21.9–120.5)	0.9606
IL-10	27.0	15.0 (8.1–30.6)	15.5	5.6 (1.5–30.8)	24.6	13.8 (5.6–30.6)	0.0551
IL-12	2.0	0.0 (0.0–1.5)	0.3	0.0 (0.0–0.0)	1.6	0.0 (0.0–1.1)	0.0123
TNF-α	5.2	1.7 (0.0–6.9)	0.2	0.0 (0.0–0.0)	4.2	0.8 (0.0–3.9)	<0.0001

Abbreviations: HIV, human immunodeficiency virus; IL, interleukin; IL-1β, interleukin-1 beta; IQR, interquartile range; pg/mL, picograms per milliliter; TNF-α, tumor necrosis factor-alpha. Notes: * *p*-values were calculated using the Wilcoxon rank-sum test to compare cytokine levels between survivors and patients who died from kala-azar. Values of 0.0 indicate concentrations below the limit of detection.

**Table 5:** Diagnostic performance of interleukin-8 (IL-8) for predicting mortality in patients with kala-azar, stratified by HIV coinfection status (total n = 90).

Parameter	n	AUC (95 % CI)	* *p*-Value (DeLong Test)
IL-8 in patients without HIV	71	0.79 (0.67–0.89)	–
IL-8 in patients with HIV	19	0.77 (0.51–1.00)	0.9216

Abbreviations: AUC, area under the receiver operating characteristic (ROC) curve; HIV, human immunodeficiency virus; IL-8, interleukin-8. Notes: * *p*-values were calculated using the DeLong test to compare the AUC of IL-8 across HIV coinfection strata. The sample size (n) for each subgroup represents the number of patients with valid IL-8 measurements included in the ROC analysis.

## Data Availability

The original contributions presented in this study are included in the article material. Further inquiries can be directed to the corresponding author.

## Abbreviations

The following abbreviations are used in this manuscript:

AUC	Area under the curve
CI	Confidence interval
CNPq	Conselho Nacional de Desenvolvimento Científico e Tecnológico
HIV	Human immunodeficiency virus
IFN-γ	Interferon-gamma
IL	Interleukin
IL-1β	Interleukin-1 beta
ROC	Receiver operating characteristic curve
TNF-α	Tumor necrosis factor-alpha
